# Common domains of nurses’ competencies in public health emergencies: a scoping review

**DOI:** 10.1186/s12912-023-01655-5

**Published:** 2023-12-21

**Authors:** Xue-E. Guo, Li-Fang Bian, Yan Li, Chun-Yan Li, Yu Lin

**Affiliations:** 1 Department of Nursing, The First Affiliated Hospital, Zhejiang University School of Medicine, Hangzhou, 310003 Zhejiang Province China; 2 Department of Nursing, Department of Hepatobiliary and Pancreatic Surgery, The First Affiliated Hospital, Zhejiang University School of Medicine, No. 79 Qing Chun Road, Hangzhou, 310003 Zhejiang Province China

**Keywords:** Public health emergencies, Nurse, Competency

## Abstract

**Background:**

A public health emergency can cause large numbers of deaths in a short period, with devastating social, economic and health consequences. Nurses are the main healthcare providers during such emergencies, and their competencies affect the control and outcomes of the situation. Studies on nurses’ competencies in public health emergencies vary between countries and healthcare systems. Therefore, we conducted a scoping review to identify the common domains of nurses’ competencies in public health emergencies worldwide.

**Methods:**

We searched the PubMed, CINHAL, Scopus, Web of Science, Science Direct, Embase, Cochrane Library, WanFang and ECRI databases from their inception to 2023. All published articles on nurses’ competencies in public health emergencies that were published in English and Chinese were included. We mainly analyzed and synthesized nurses’ competencies, assessment instruments and the training described in the included studies.

**Results:**

A total of 27 competency domains were identified following an analysis and summary. The most frequently cited domains were communication skills, self-protection skills, basic knowledge of a public health emergency, laws and ethics and the capacity for organizational collaboration. The Disaster Preparedness Evaluation Tool and the Emergency Preparedness Information Questionnaire were the most commonly used tools for assessing competencies. Most training was conducted online and the content that was covered varied by country.

**Conclusions:**

Given the significant roles and responsibilities of nurses in public health emergencies, knowing the domains of their competencies is essential to evaluating, developing, and conducting clinical training.

**Supplementary Information:**

The online version contains supplementary material available at 10.1186/s12912-023-01655-5.

## Background

A public health emergency is defined as the occurrence or imminent threat of a disease or health condition (e.g., an infectious disease or bioterrorist attack) that poses a significant risk of death, injury or long-term or permanent disability in a large number of people [1]. A public health emergency has the potential to cause large numbers of deaths in a short period of time, with devastating social, economic and health consequences [2]. It challenges the preparedness and capacity for responses by governments, hospitals, clinics, public health institutions and academic researchers [3]. How to respond to a public health emergency has become a vital issue for governments and international organizations worldwide [4].

Once a public health emergency occurs, the health department must initiate a medical rescue quickly and efficiently to minimize casualties and health hazards. Nurses have multiple roles and responsibilities in public health emergencies; they work with limited resources in fast-paced environments and perform critical tasks, such as triage and first aid [5, 6]. It is challenging for inexperienced nurses to be involved in rescues without training.

Competency is defined as a combination of the knowledge, skills and abilities required to perform a specific task [7]. Determining nurses’ competencies would be helpful for making preparations to provide training and to conduct research [8]. Several countries have formulated competency sets in accordance with the characteristics of their healthcare system and the types of public health emergencies or disasters. In the United States (US), hurricanes, terrorist attacks and bioterrorism have increased attention to disasters; hence, studies have focused on the competencies associated with disasters. In China, most studies have focused on infectious diseases because of the outbreak of major infectious diseases.

Two reviews have outlined nurses’ competencies in disasters [9, 10], but both of them only described the competencies of each of the included studies, and did not group. These competency domains might not have been sufficient or specific to encounters with infectious disease outbreaks. The outbreak of COVID-19 highlighted the importance of enhancing nurses’ competencies [11–13], many studies have been conducted to identify competencies that should be required [14–16]. Thus, it was vital to conduct a scoping review to analyze and synthesize the existing research to identify the most common domains of nurses’ competencies during public health emergencies with the intent of improving formal education and training programs for nurses.

## Methods

### Design

A scoping review method was used, incorporating explanations, interpretations, and summaries of quantitative and qualitative literature to address research questions. This approach allows for review to extract different data and develop them in a meaningful, transparent, and systematic way [17]. The iceberg model was used as a theoretical basis, guiding the competency domain identification process [18]. The PRISMA-ScR (Preferred Reporting Items for Systematic Reviews and Meta-analyses Extension for Scoping Reviews) checklist was used to guide the writing of this review [19].

### Search method

We searched the PubMed, CINHAL, Scopus, Web of Science, Science Direct, Embase, Cochrane Library, WanFang and ECRI databases. The search for grey literature included the BASE and Opengrey dababases. The search terms and Boolean strings used in PubMed are presented in Table 1.

**Table 1:** The search terms and Boolean strings used in the PubMed database

	Search strategy
PUBMED	("competency*"[Title/Abstract] OR "competence"[Title/Abstract] OR "capacity"[Title/Abstract] OR "ability"[Title/Abstract] OR "skill"[Title/Abstract]) AND ("public health emergency"[Title/Abstract] OR "infectious disease"[Title/Abstract] OR "bioterrorist attacks"[Title/Abstract] OR "pandemic"[Title/Abstract] OR "Ebola"[Title/Abstract] OR "COVID-19"[Title/Abstract] OR "Influenza A"[Title/Abstract] OR "Zika"[Title/Abstract] OR "Monkeypox"[Title/Abstract] OR "MERS-CoV"[Title/Abstract] OR "SARS"[Title/Abstract] OR "Yellow Fever"[Title/Abstract] OR "Smallpox"[Title/Abstract] OR "Poliovirus"[Title/Abstract] OR "Dengue"[Title/Abstract] OR "Anthrax"[Title/Abstract]) AND ("nurs*"[MeSH Terms] OR "nursing personnel"[Title/Abstract] OR "medical staff"[Title/Abstract] OR "medical worker"[Title/Abstract] OR "health professional"[Title/Abstract] OR "health workforce"[Title/Abstract] OR "healthcare worker"[Title/Abstract] OR "health worker"[Title/Abstract])

Articles that met the criteria for inclusion in the scoping review: (1) were published from the inception of the database to 2023, (2) were written in English or Chinese, (3) consisted of qualitative and quantitative studies, policy documents and grey literature and (4) focused on nurses’ competencies that were developed or described in response to public health emergencies. Articles with the following characteristics were excluded from the scoping review: (1) the full text was unavailable, (2) the study did not focus on nurses (i.e., doctors, laboratory engineers, pharmacist) or (3) the study did not address competencies.

### Search outcomes

**Fig. 1:**
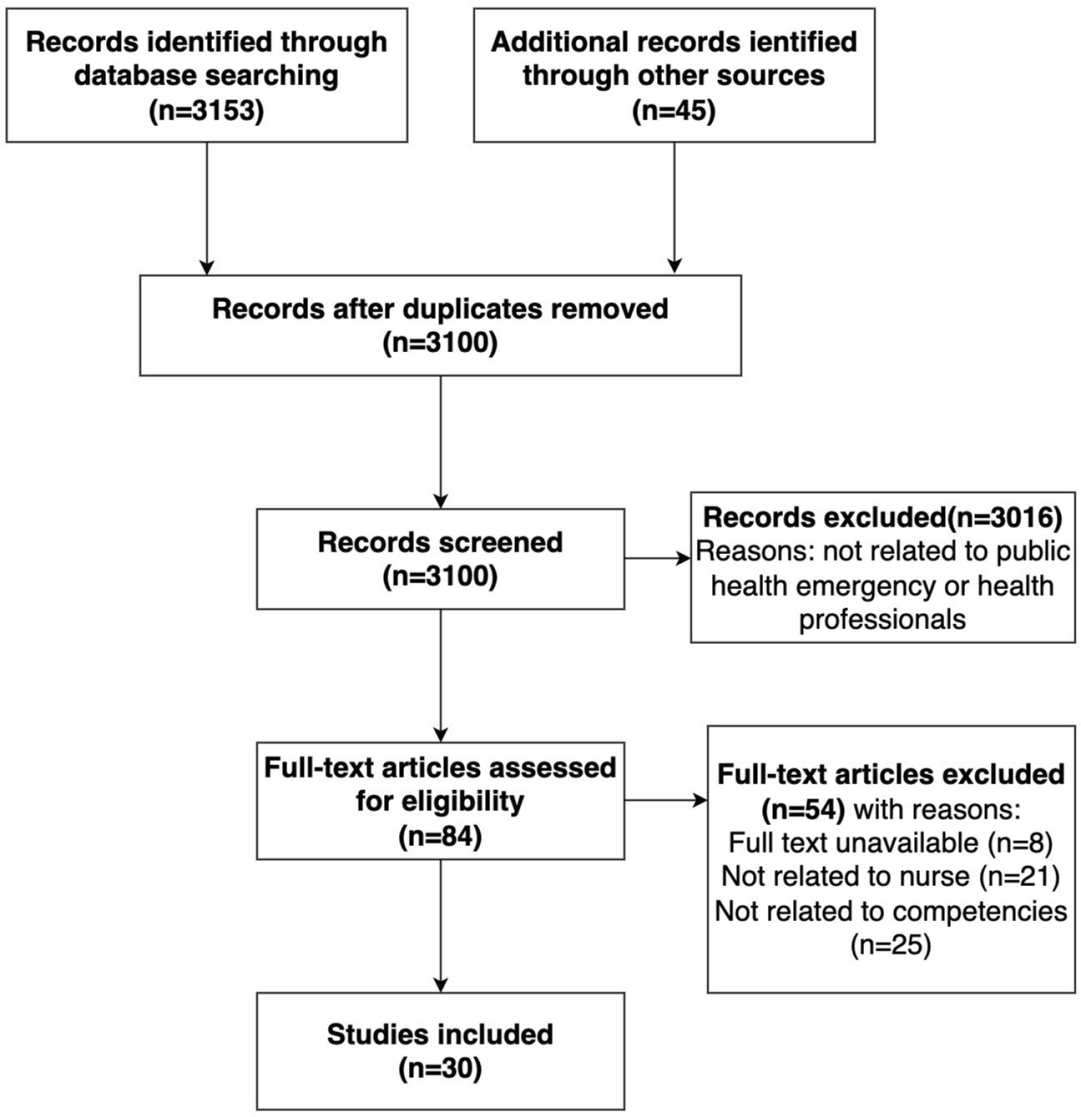
PRISMA flow chart of the selection of articles

Figure 1 depicts the process of selecting the articles for this scoping review. A total of 3153 titles were identified through a database search and other sources. After we screened the titles and abstracts, 84 studies were retained for a full-text review, of which 30 were included in the scoping review. All of the studies were read and screened independently by two of the study’s authors, in accordance with the inclusion and exclusion criteria, and disagreements were resolved by consensus or by a third author.

### Quality appraisal

The Joanna Briggs Institute Critical Appraisal Checklist for text and opinions [15], reporting prevalence data [20], quasi-experimental studies [21], cohort studies [22] and qualitative research [23] were used to conduct a quality assessment of the scoping review. Two reviewers independently assessed each included study, and any discrepancies in scores were resolved by a third reviewer. All of the articles were included in the scoping review after a quality appraisal was conducted. The results are presented in Additional file 1: Appendix I Table A I-V.

### Data extraction

The extracted data consisted of information about the studies, including the country where the research was conducted, competencies, models and methods of competencies, instruments and training, etc. Data were analyzed using Microsoft Excel and we used the following procedure to identify competency domains. First, we developed an initial set of competency domains. All of the reviewers listed competency domains independently after reading all of the included studies. We retained the identical domains, discussed different domains and eventually developed an initial set of competency domains. Second, two of the reviewers independently listed all of the competencies mentioned in all of the included studies; any disagreement was resolved through consensus or by a third reviewer. Third, two reviewers coded studies and competencies to identify them. We encoded the selected studies using the letter "S" followed by three numbers, with "S001" as the code to identify the first study. We encoded the agreed upon competencies as the letter “C” followed by three numbers, with “C001” as the code to identify the first competency (e.g., "C123 S010" could be identified as competency 123 belonging to study 10). After a group discussion, we assigned all competencies to the specific domain with which they most closely aligned and considered whether we needed to make changes to the initial set of competency domains. The frequency of each competency domain was counted to identify the domain with the most competencies.

## Results

The 30 papers that were examined in this scoping review were conducted in 12 countries: China, the US, India, Korea, Syria, South Africa, England, Turkey, Kenya, Canada, Slovenia and Israel. The characteristics of the included studies are presented in Tables 2, 3 and 4

**Table 2:** Summary of the identified competencies in the included studies

Study	Outcome	Approach
(Bai et al., 2022) [24]	Constructed a competency evaluation index system of front-line nurses, including 64 competency indicators	Literature review, semi-structured in-depth interviews, and the Delphi approach
(Cui et al., 2022) [25]	Developed a competency model to combat public health emergencies, including 30 items in four dimensions	Literature review, key informant interviews, the Delphi approach and questionnaire survey
(Dashash et al., 2020) [16]	Identified 52 competencies required for approaching patients with COVID-19	The Delphi technique and virtual meeting
(Mao, et al., 2021) [26]	Reported the action and incident management of nurse, and the core competencies in ICN CCDN V2.0	Cite ICN CCDN V2.0
(Papadopoulos, 2022) [27]	Described the importance of cultural competence	/
(Wu et al., 2021) [14]	Established a core competency evaluation index system for infectious diseases specialist nurses, including 47 indexes	Literature review, qualitative face-to-face interviews, and Delphi approach
(Zhao et al., 2022) [28]	Developed and validated a model of public health emergency competency, including 43 items	Literature review, expert consultation, critical events and focus group interview
(Qiao & Yang, 2014) [29]	Analyzed the response competency requirement of public health emergencies for emergency specialized nurses and constructed an evaluation system for the competency, including 36 items	Delphi approach
(Bian et al., 2021) [30]	Formulated a competency index system of nurses in public health emergencies based on the Iceberg Model, including 27 indicators	Literature review and Delphi approach
(Lin et al., 2021) [31]	Constructed an index system for community nurses' emergency public health emergency rescue capacity, including 51 competencies	Literature review, qualitative interview and Delphi approach
(Kan et al., 2018) [32]	Established a core response competency index system for infectious disease emergencies, and comprised 38 indexes	Literature review and Delphi approach
(Huang et al., 2021) [33]	Established the core competency index system for emergency rescue of infectious diseases among military advance nurses, including 52 indicators	Literature review and modified Delphi approach
(Chan et al., 2011) [34]	Drafted the core competency items of hospital infection control nurses, including 51 items	Literature review and Delphi approach
(Jorgensen et al., 2010) [35]	Adopted core competency set based on national consensus for perinatal and neonatal nurses to guide emergency preparedness and disaster education and training	Based on national consensus
(Polivka et al., 2008) [36]	Developed consensus on public health nursing competencies in public health surge events	Literature review, Delphi approach and focus group

**Table 3:** Summary of the included studies on training

Author/Year	Competency	Content	Results	Approach
(Slobodin et al., 2021) [37]	Cultural competency	The historical view and negative impact of pandemics; the challenges of health services; the importance of cultural competency; culturally competent care, attitudes, skills and healthcare team in COVID-19	Online program can improve nurses’ competence, and the highest gains were in the attitude domains, whereas the lowest in the knowledge domain	website
(McGarity et al., 2022) [15]	Self-protection skills; Emergency response skills; First aid skills; Basic nursing skills;	Mock code blue of an infectious patients; personal protective equipment practice; triaging practice and bundling of care	Just-in-time education is a good training strategy	Just-in-time education strategy
(Zhang et al., 2021) [38]	Self-protection skills; Critical care skills;	Pre-hospital management; fever clinic reception; intensive care unit; isolation ward training	Knowledge, the capacity of emergency care and disaster preparedness of nurses increased after intervention. The technical skills improved more in group used virtual reality simulation	Virtual reality simulation training
(Qureshi et al., 2002) [39] (Qureshi, 2004) [40]	1. Policy and regulations 2. Communication skills 3. Critical thinking skills 4. Monitoring capacity	Definition of Emergencies and disasters; The role and responsibilities of agencies in emergencies; The emergency response command system and emergency response protocols; The public health nurses’ role; Emergency communication abilities and the Use of related special equipment; Shelter management procedures	Basic emergency preparedness training can help health department achieve consistent and positive employee response to emergencies	Program slides and brochure in (Qureshi, 2004)
(Jen et al., 2022) [41]	Self-protection skills	proper sequence and techniques for donning and doffing PPE	The mobile video online learning method reduced learning anxiety, improved COVID-19 prevention knowledge, academic performance, critical thinking ability, and learning self-efficacy of nurses	Mobile-video online learning
(Dhal & Mohapatra, 2022) [42]	1. Communication skills 2. Professional development capacity 3. Basic nursing skills 4. Mentoring skills 5. Adaptation 6. Ethical skills	/	Identifying training need help nurse perform better during pandemic	/
(Lavin et al., 2019) [43]	1. Preparation 2. Communication skills 3. Self-protection skills 4. Laws and ethics 5. Policy and regulations	The definition of vector; Building community action plan; Success story	The online learning tool kits can increase nurses’ knowledge of public health emergencies, and improved preparedness	Platform: Thing Link
(Lauck et al., 2022) [44]	Critical care skills	The basics of mechanical ventilation; foundations of acute/critical care practice,; electrocardiogram interpretation; oxygenation and ventilation and shock states	Nurses reported a high- level of perceived support and safe care provided	Remote education modules

**Table 4:** Summary of the cross-sectional included studies

Author/Year	Instrument	Characteristics	Results
(Alan et al., 2022) [45]	Nurses’ Perceptions of Disaster Core Competencies Scale	The scale consists of 5 subscales, a total of 45 items (critical thinking skills, special diagnostic skills, general diagnostic skills, technical skills, and communication skills). The response ranging from 1 to 5 point. And the Cronbach’s **α** is 0.96	The level of disaster core competency of nurses was higher than average. It was positively correlated with psychological resilience and had a strong correlation with disaster experience
(Hong, Jung, & Woo, 2022) [46]	The Korean version of the Disaster Preparedness Evaluation Tool	28 items were scored on a 6-point Likert scale, including disaster education training, disaster knowledge and information; bioterrorism and emergency response; and disaster assessment. The Cronbach’s alpha of DPET-K is 0.954	The average score of disaster competencies was 84.08 ± 24.74 out of 168, and the prevention stage was the highest
(Jang, Kim, & Lee, 2022) [47]	the Disaster Nursing Preparedness-Response Competency	The scale focus on the disaster nursing preparedness competency and disaster nursing response competency, including 34 items, and each item rating on five-point. The Cronbach’s alpha is 0.96, and the reliability is 0.91–0.92 in this study	The mental health nurses’ disaster nursing competencies scores was 115.12 ± 16.38 out of 170. The influencing factors included compassion satisfaction, disaster nursing experience, and disaster nursing training
(Karnjuš, Prosen, & Ličen, 2021) [48]	the Slovenian version of the Disaster Nursing Core Competencies Scale	The scale is 7-point Likert, consisting of 39 items, nurses’ core competencies in disaster management (27 items), barriers to developing core competencies (8 items), and the role and responsibilities of nurses in disaster management (4 items). And the Cronbach α is 0.937	Registered nurses considered core competencies in disaster nursing critical to their preparation for disaster situations. Nurses working in nursing homes and nursing managers are more aware of acquiring the listed competencies for unexpected events and their role in disaster management
(Li et al., 2021) [49]	The core emergency response competency questionnaire	The scale included two parts, basic characteristics of participants and knowledge about COVID-19 (prevention, emergency preparedness and emergency rescue competencies). The content validity index is 0.870, and the Cronbach’s α is 0.957	Nurses had an excellent grasp of COVID-19, but most needed to gain experience in isolation ward work and emergency training
(Song, Li, Bell, Yang, & Zhang, 2021) [50]	Modified core emergency response competency questionnaire	The scale included preventive ability, preparation ability and rescue ability, a total of 36 items, and is scored at 5 levels using the Likert scale. The Cronbach’s α of the scale is 0.97, and the content validity index is 0.87	Nurses' core emergency response ability for major infectious diseases was at a medium level, and the influencing factors were infectious disease emergency drills, academic qualifications, and work experience in infectious disease nursing

### Identified competencies

We identified 590 competency indicators by analyzing and summarizing the competencies mentioned in the included studies, and we sorted them into 27 competency domains. Based on the iceberg model, the competency domains were divided into three major dimensions: knowledge, skills, and personal characteristics (Table 5).

**Table 5:** Competency domains

	Domains	Studies	Frequency
Knowledge	Basic knowledge of public health emergencies	(Bai et al., 2022) [24]; (Dashash, Almasri, Takaleh, Halawah, & Sahyouni, 2020) [16]; (Wu et al., 2021) [14]; (Qiao & Yang, 2014) [29]; (Bian, Yu, Liu, & Bai, 2021) [30]; (Kan T, 2018) [32]; (Huang, Zhou, & Ma, 2021) [33]; (Polivka et al., 2008) [36]; (Slobodin, Kula, Clempert, & Cohen, 2021) [37]	9
	Laws and ethics	(Bai et al., 2022) [24]; (Mao et al., 2021) [26]; (Qiao & Yang, 2014) [29]; (Bian et al., 2021) [30]; (Kan T, 2018) [32]; (Jorgensen, Mendoza, & Henderson, 2010) [35]; (Polivka et al., 2008) [36]; (Lavin et al., 2019) [43] (Dhal & Mohapatra, 2022) [42]	9
	Policy and regulations	(Dashash et al., 2020) [16]; (Bian et al., 2021) [30]; (Kan T, 2018) [32]; (Polivka et al., 2008) [36]; (Lavin et al., 2019) [43]; (Qureshi et al., 2002) [39]; (Qureshi et al., 2004) [40]	7
	Contingency plans	(Cui et al., 2022) [25]; (Mao et al., 2021) [26]; (Bian et al., 2021) [30]; (Jorgensen et al., 2010) [35]; (Polivka et al., 2008) [36]	5
Skills	Communication skills	(Bai et al., 2022) [24]; (Dashash et al., 2020) [16]; (Mao et al., 2021) [26]; (Wu et al., 2021) [14]; (Qiao & Yang, 2014) [29]; (Bian et al., 2021) [30]; (Lin, Zhu, & Chen, 2021) [31]; (Jorgensen et al., 2010) [35]; (Polivka et al., 2008) [36]; (Dhal & Mohapatra, 2022) [42]; (Lavin et al., 2019) [43]; (Qureshi et al., 2002) [39]; (Qureshi et al., 2004) [40]	13
	Self-protection skills	(Bai et al., 2022) [24]; (Cui et al., 2022) [25]; (Mao et al., 2021) [26]; (Wu et al., 2021) [14]; (Bian et al., 2021) [30]; (Jorgensen et al., 2010) [35]; (McGarity et al.,) [15]; (Zhang et al., 2021) [38]; (Jen, Chou, & Chang, 2022) [41]; (Lavin et al., 2019) [43]	10
	Capacity for organizational collaboration	(Bai et al., 2022) [24]; (Cui et al., 2022) [25]; (Wu et al., 2021) [14]; (Qiao & Yang, 2014) [29]; (Bian et al., 2021) [30]; (Lin et al., 2021) [31]; (Chan, Adamson, Chung, & Chow, 2011) [34]; (Polivka et al., 2008) [36]	8
	Reporting and notification skills	(Cui et al., 2022) [25]; (Wu et al., 2021) [14]; (Bian et al., 2021) [30]; (Lin et al., 2021) [31]; (Kan T, 2018) [32]; (Jorgensen et al., 2010) [35]; (Polivka et al., 2008) [36]	7
	Assessment & diagnostic skills	(Cui et al., 2022) [25]; (Dashash et al., 2020) [16]; (Mao et al., 2021) [26]; (Zhao et al., 2022) [28]; (Bian et al., 2021) [30]; (Lin et al., 2021) [31]; (Polivka et al., 2008) [36]	7
	Crisis intervention skills	(Bai et al., 2022) [24]; (Dashash et al., 2020) [16]; (Papadopoulos, 2022) [27]; (Qiao & Yang, 2014) [29]; (Bian et al., 2021) [30]; (Lin et al., 2021) [31]; (Polivka et al., 2008) [36]	7
	First aid skills	(Zhao et al., 2022) [28]; (Qiao & Yang, 2014) [29]; (Bian et al., 2021) [30]; (Lin et al., 2021) [31]; (Huang et al., 2021) [33]; (Polivka et al., 2008) [36]; (McGarity et al., 2022) [15]	7
	Management	(Cui et al., 2022) [25]; (Dashash et al., 2020) [16]; (Mao et al., 2021) [26]; (Qiao & Yang, 2014) [29]; (Bian et al., 2021) [30]; (Chan et al., 2011) [34]	6
	Capacity for professional development	(Cui et al., 2022) [25]; (Wu et al., 2021) [14]; (Qiao & Yang, 2014) [29]; (Bian et al., 2021) [30]; (Chan et al., 2011) [34]; (Dhal & Mohapatra, 2022) [42]	6
	Critical thinking skills	(Bai et al., 2022) [24]; (Wu et al., 2021) [14]; (Qiao & Yang, 2014) [29]; (Bian et al., 2021) [30]; (Qureshi et al., 2002) [39]; (Qureshi et al., 2004) [40]	6
	Monitoring capacity	(Cui et al., 2022) [25]; (Kan T, 2018) [32]; (Chan et al., 2011) [34]; (Polivka et al., 2008) [36]; (Qureshi et al., 2002) [39]; (Qureshi et al., 2004) [40]	6
	Preparation	(Mao et al., 2021) [26]; (Bian et al., 2021) [30]; (Lin et al., 2021) [31]; (Jorgensen et al., 2010) [35]; (Lavin et al., 2019) [43]	5
	Basic nursing skills	(Bai et al., 2022) [24]; (Wu et al., 2021) [14]; (Polivka et al., 2008) [36]; (McGarity et al., 2022) [15]; (Dhal & Mohapatra, 2022) [42]	5
	Critical care skills	(Bai et al., 2022) [24]; (Qiao & Yang, 2014) [29]; (Wu et al., 2021) [14]; (Zhang et al., 2021) [38]; (Lauck et al., 2022) [44]	5
	Guard and security capacity	(Bai et al., 2022) [24]; (Bian et al., 2021) [30]; (Huang et al., 2021) [33]; (Jorgensen et al., 2010) [35]	4
	Health education skills	(Cui et al., 2022) [25]; (Bian et al., 2021) [30]; (Lin et al., 2021) [31]; (Polivka et al., 2008) [36]	4
	Cultural competencies	(Papadopoulos, 2022) [27]; (Jorgensen et al., 2010) [35]; (Slobodin et al., 2021) [37]	3
Personal characteristics	Responsibility	(Bai et al., 2022) [24]; (Cui et al., 2022) [25]; (Qiao & Yang, 2014) [29]; (Bian et al., 2021) [30]	4
	Stress coping skills	(Bai et al., 2022) [24]; (Cui et al., 2022) [25]; (Zhao et al., 2022) [28]	3
	Adaptation	(Bian et al., 2021) [30]; (Dhal & Mohapatra, 2022) [42]	2
	Spirit of dedication	(Bai et al., 2022) [24]; (Cui et al., 2022) [25]	2
	Optimism	(Bai et al., 2022) [24]; (Cui et al., 2022) [25]	2
	Empathy	(Dashash et al., 2020) [16]	1

After counting the frequency of the competency domains mentioned in the studies, the five most-cited competency domains were found to be communication skills, self-protection skills, basic knowledge of public health emergencies, laws and ethics and capacity for organizational collaboration. Self-protection skills mainly included the proper use of personal protective equipment (PPE), hand hygiene, infection control principles and medical waste disposal skills. Effective communication with physicians, patients and their families were expected of nurses, in addition to a basic knowledge of public health emergencies, mainly including definitions, categories, etiology, epidemiology, prevention and control. Issues related to laws and ethics were attended to within the legal and ethical framework of public health emergencies. The capacity for organizational collaboration included coordination, teamwork, collaboration and organization.

Literature reviews, in-depth interviews, key informant interviews, questionnaire surveys and the Delphi approach were used to identify competencies, of which the Delphi technique was used most often. Related theories included the iceberg theory, the onion theory, the Miller hierarchy, the Prevention, Preparedness, Response and Recovery Model (PPRR Model), the Three-phase Emergency Response Theory and the World Health Organization framework for taking action on infectious disease outbreaks. The PPRR Model was used most often.

### Assessment instruments

The instruments used to assess nurses’ competencies during public health emergencies were as follows:

1. the Nurses’ Perceptions of Disaster Core Competencies Scale (NPDCC);
2. the Korean version of the Nurse Disaster Preparedness Evaluation Tool (DPET-K), which was adapted from the DPET [51, 52];
3. the Disaster Nursing Preparedness-Response Competency (DNPRC) score, which was based on the International Council of Nurses’ Core Competencies in Disaster Nursing, version 1.0 (ICN CCDN V1.0) [53];.
4. the Slovenian version of the Disaster Nursing Core Competencies Scale (SL-DNCC-Scale). The original DNCC was designed by Abdulellah Al Thobaity [54, 55];
5. the Core Emergency Response Competency Questionnaire developed by Kan Ting [32]; and.
6. the EPIQ, which was designed by the Wisconsin Nurses’ Association [56].

The DPET and EPIQ were the most commonly used assessment tools; they were widely used in different countries. The NPDCC was used in Turkey, Iran and China and the DNPRC was used in Korea though less often. The DNCC was used in the Kingdom of Saudi Arabia and Slovenia, and the Core Emergency rResponse Competency Questionnaire was widely used in China.

### Training

Training can prepare nurses for future public health emergencies by helping them improve their competencies and acquire new ones. The focus of training is different in each country due to differences in their healthcare systems and cultures. The basic training curricula for public health emergencies in the US focused on emergency preparedness for public health nurses and was introduced in 2002. However, training in China mostly began during the COVID-19 outbreak and focused on nurses' professional and technical skills, especially critical care skills and knowledge of PPE. Most of the training, which was conducted online used relevant training methods, including virtual reality simulation and mobile-video online learning. Just-in-time education was also a common training strategy. It is an educational method based on work, in which a person is trained at a time that is close to the actual clinical event [57]. Rapid cycle deliberate practice includes immediate directional feedback, which allows brief corrective instructions to be given to the learner and is followed by a repeated attempt of the learner to master the learning task [58].

## Discussion

Until now, no unified standards have been formulated for nursing competencies in public health emergencies. However, this scoping review focused on identifying nurses’ competencies regarding public health emergencies and summarizing relevant evaluation instruments and training practices to improve formal education and training programs for nurses.

### Identification of common domains of competencies

The most-cited competency domains in this scoping review were communication skills, self-protection skills, basic knowledge of public health emergencies, laws and ethics, and the capacity for organizational collaboration. Hospitals often exhibit poor communication as well as a lack of planning, empowerment, motivation, a common language, and emergency-trained nurses [59]. Effective communication is crucial for supporting nurses through extended periods of crisis, and relevant communication strategies should be developed [60–62]. To prevent the spread of infectious diseases in pandemics, it is vital for nurses to implement protective measures in order to reduce infection risk [63]. During SARS-Cov-2, training and demonstrations for donning and doffing PPE safely was the best way for nurses to prevent infection [64]. Therefore, there is a need to strengthen the training of healthcare workers to prepare them for the next outbreak. The basic knowledge of public health emergencies mainly include disease-related epidemiology: etiology of disease, origin, incubation period and transmission; quarantine, contacts and contact tracing principles; related diagnostic tests; signs and symptoms of disease; infection control and prevention strategies; and evidence-based drug use. Nurses are required to know and abide by the laws regarding professional responsibility, licensure, and volunteering for public health emergencies. Ethics and value as a concept was the core element in the resilience framework for public health emergency preparedness [65]. It was essential for a country to respond effectively to public health emergency [66–68]. According to the American Nurses Association’s Code of Ethics for Nurses with Interpretive Statements, nurses are expected to practice with compassion and respect for patients and to commit to them [69]. A public health emergency rescue usually relies on an interdisciplinary team and multisectoral collaboration, with the capacity for good organizational collaboration that can improve rescue efficiency [70, 71].

Researchers often use methods and theoretical frameworks to develop a competency set. The Delphi method, which has been widely used in nursing practice, was a useful tool for identifying competencies because it promotes ownership and increasing acceptance of the consensus generated by the process [72–74]. The Delphi method, which was used in many of the included studies established a panel of experts, and conducted anonymous surveys on specific topics using structured questionnaires in successive rounds. And researchers analyzed and amended the content based on expert recommendations until consensus was reached [75]. A comprehensive approach to the identification of competencies was often used, based on the previous literature, which combined Delphi methods with interviews to ensure the integrity and scientific accuracy of the information. The research areas of experts should include public health, infectious diseases, critical care, medicine, emergency nursing and nursing management. The experts who participated in public health emergencies are preferred. Interviews may involve specialists, nurses or patients. The steps for identifying competencies in China were to: choose a theoretical framework, analyze and summarize the literature and qualitative interviews; refine the entries and formulate the initial competency set; make revisions using Delphi expert consultation and reach a consensus to finalize the competency set. The process in the US involved using the Delphi method to identify competencies, and then conducting a focus group to assess the identified competencies. Competencies were primarily determined by researchers or clinicians in China, and by associates in the US. The competency model was often based on a theoretical framework, and the PPRR Model was the most cited framework [76]. The PPRR model was used in disaster risk reduction and emergency management, outlining various stages of the disaster cycle [77]. In this review, we used the iceberg model to synthesize identified competency because the personal characteristics were not included in the PPRR model. The iceberg model is also widely used to develop nurse competency model [78, 79].

### Nurses’ training programs based on emergency competencies

Current training is mainly competency-based and focuses on nurses’ knowledge and skills in public health emergencies. Since the outbreak of COVID-19, recent studies have shown that an increasing amount of attention has been paid to the cultivation of culture and personal characteristics competencies, which is consistent with the iceberg model of competency. Increasing cultural training will help promote nurses’ initiative and willingness to provide nursing care during public health events. Personal characteristics, social roles and values, which play key roles in distinguishing individual behavior and performance, have often been ignored. Incorporating personal characteristics into assessments and training may help select nurses who are more suitable for front-line rescues and for practicing scientific competency-based human resource management. China has a collectivist culture, which emphasizes cohesion, duty and the achievement of group goals. Therefore, Chinese nurses have a high level of willingness to respond to public health emergencies and some Chinese nurses even believe they lived out their calling during COVID-19 [80].

Some of the assessment instruments investigated in this review can be used to evaluate nurses' competencies and to develop relevant training. The DPET and EPIQ were the two most commonly used evaluation tools. The items of the DPET were used to assess the competencies of the nurses in at three disaster stages: the pre-disaster, mitigation and response and evaluation stages. The DPET has been translated and used in many countries, including Korea, Japan, Indonesia, China, Iran, Jordan, Thailand and Saudi Arabia [46, 81–88]. Some of the studies verified the validity and reliability of these instruments in their respective countries [81, 83, 86]. The EPIQ consists of two parts: the dimensions of personal information and disaster preparedness competencies. This instrument has also been used in other countries. (e.g., the United Kingdom, Malaysia, Saudi Arabia, Iran and Korea [89–93].

### Implications for nursing management

Nursing managers should emphasize improvements in nurses’ competencies in public health emergencies, strengthen relevant training and prepare for the next epidemic. Our identification of competency domains in this scoping review will foster the development of an education curriculum or clinical training program. Training methods and strategies that were synthesized in this scoping review can be used in clinical training to help nurses quickly master relevant skills, and the instruments for evaluating competencies can help nurse managers select competent nurses, ensure a high quality of care, provide feedback on training results and adjust relevant training programs.

## Limitations

Conceptual limitations: this scoping review focused on nurse competency in infectious disease outbreaks, with relatively few studies on other disasters. Language biases: the languages of the included studies were limited to English and Chinese; hence, it is also necessary to determine the competencies reported in studies published in different languages. Implicit biases: the cultural backgrounds of the researchers may have influenced their perspectives.

## Conclusion

This scoping review outlined the common domains of nurses’ competencies in public health emergencies. Three dimensions with 27 competency domains were identified after they were analyzed and synthesized, and the most-cited competency domains were self-protection skills, communication skills, basic knowledge of public health emergencies, laws and ethics and capacity for organizational collaboration. The identified competencies may be helpful for developing an education curriculum and for conducting clinical training. The competency assessment instruments, training methods, and strategies synthesized in this scoping review will be useful for nursing management and future research.

### Supplementary Information

**Additional file 1: Table A 1.** JBI critical appraisal checklist for text and opinion papers. **Table A 2.** Quality assessment of opinion papers. **Table A 3.** JBI critical appraisal checklist for studies reporting prevalence data. **Table A 4.** Quality assessment of descriptive studies. **Table A 5.** JBI critical appraisal checklist for quasi-experimental studies. **Table A 6.** Quality assessment of quasi-experimental studies. **Table A 7.** JBI critical appraisal checklist for cohort studies. **Table A 8.** Quality assessment of cohort study. **Table A 9.** JBI critical appraisal checklist for qualitative research. **Table A 10.** Quality assessment of qualitative studies.

## Data Availability

All data generated or analysed during this study are included in this published article.

## Abbreviations

COVID-19: Coronavirus disease
DNPRC: Disaster Nursing Preparedness-Response Competency score
DPET: Nurse Disaster Preparedness Evaluation Tool
DPET-K: Korean version of the Nurse Disaster Preparedness Evaluation Tool
EPIQ: Emergency Preparedness Information Questionnaire
ICN CCDN V1.0: International Council of Nurses Core Competencies in Disaster Nursing, version 1.0
NPDCC: Nurses’ Perceptions of Disaster Core Competencies Scale
PPE: Personal protective equipment
PPRR Model: Prevention, Preparedness, Response and Recovery Model
PRISMA-ScR: Preferred Reporting Items for Systematic Reviews and Meta-analyses Extension for Scoping Reviews
SARS-Cov-2: Severe Acute Respiratory Syndrome Coronavirus-2
SL-DNCC-Scale: The Slovenian version of the Disaster Nursing Core Competencies Scale
US: United States
