# Supplementary material for: Common domains of nurses’ competencies in public health emergencies: a scoping review
Source: BMC Nurs. 2023 Dec 21;22:490. doi: 10.1186/s12912-023-01655-5 (PMC10734140; doi:10.1186/s12912-023-01655-5)
Supplement: Supplementary file 1 — Additional file 1: Table A 1. JBI critical appraisal checklist for text and opinion papers. Table A 2. Quality assessment of opinion papers. Table A 3. JBI critical appraisal checklist for studies reporting prevalence data. Table A 4. Quality assessment of descriptive studies. Table A 5. JBI critical appraisal checklist for quasi-experimental studies. Table A 6. Quality assessment of quasi-experimental studies. Table A 7. JBI critical appraisal checklist for cohort studies. Table A 8. Quality assessment of cohort study. Table A 9. JBI critical appraisal checklist for qualitative research. Table A 10. Quality assessment of qualitative studies. [file 12912_2023_1655_MOESM1_ESM.docx]

**Appendix** **Ⅰ Literature quality evaluation results**

**Table A 1** JBI critical appraisal checklist for text and opinion papers

| **Domain** | **Indicator** |
| --- | --- |
| Q1-Is the source of the opinion clearly identified? | Yes-No-Unclear-Not applicable |
| Q2-Does the source of opinion have standing in the field of expertise? | Yes-No-Unclear-Not applicable |
| Q3-Are the interests of the relevant population the central focus of the opinion? | Yes-No-Unclear-Not applicable |
| Q4-Is the stated position the result of an analytical process, and is there logic in the opinion expressed? | Yes-No-Unclear-Not applicable |
| Q5-Is there reference to the extant literature? | Yes-No-Unclear-Not applicable |
| Q6-Is any incongruence with the literature/sources logically defended? | Yes-No-Unclear-Not applicable |

**Table A 2** Quality assessment of opinion papers

|  | **Q1** | **Q2** | **Q3** | **Q4** | **Q5** | **Q6** |
| --- | --- | --- | --- | --- | --- | --- |
| (Bai et al., 2022) | Yes | Yes | Yes | Yes | No | No |
| (Cui et al., 2022) | Yes | Yes | Yes | Yes | Yes | Yes |
| (Dashash et al., 2020) | Yes | Yes | Yes | Yes | Yes | No |
| (Papadopoulos, 2022) | Yes | Yes | Yes | Yes | Yes | No |
| (Wu et al., 2021) | Yes | Yes | Yes | Yes | Yes | No |
| (Zhao et al., 2022) | Yes | Yes | Yes | Yes | Yes | No |
| (Qiao & Yang, 2014) | Yes | Yes | Yes | Yes | Yes | No |
| (Bian et al., 2021) | Yes | Yes | Yes | Yes | Yes | No |
| (Lin et al., 2021) | Yes | Yes | Yes | Yes | Yes | No |
| (Kan T, 2018) | Yes | Yes | Yes | Yes | Yes | No |
| (Huang et al., 2021) | Yes | Yes | Yes | Yes | Yes | No |
| (Chan et al., 2011) | Yes | Yes | Yes | Yes | No | No |
| (Jorgensen et al., 2010) | Yes | Yes | Yes | Yes | Yes | No |
| (Polivka et al., 2008) | Yes | Unclear | Yes | Yes | Yes | No |

**Table A 3** JBI critical appraisal checklist for studies reporting prevalence data

| **Domain** | **Indicator** |
| --- | --- |
| Q1-Was the sample frame appropriate to address the target population? | Yes-No-Unclear-Not applicable |
| Q2-Were study participants sampled in an appropriate way? | Yes-No-Unclear-Not applicable |
| Q3-Was the sample size adequate? | Yes-No-Unclear-Not applicable |
| Q4-Were the study subjects and the setting described in detail? | Yes-No-Unclear-Not applicable |
| Q5-Was the data analysis conducted with sufficient coverage of the identified sample? | Yes-No-Unclear-Not applicable |
| Q6-Were valid methods used for the identification of the condition? | Yes-No-Unclear-Not applicable |
| Q7-Was the condition measured in standard, reliable way for all participants? | Yes-No-Unclear-Not applicable |
| Q8-Was there appropriate statistical analysis? | Yes-No-Unclear-Not applicable |
| Q9-Was the response rate adequate, and if not, was the low response rate managed appropriately? | Yes-No-Unclear-Not applicable |

**Table A 4** Quality assessment of descriptive studies

|  | **Q1** | **Q2** | **Q3** | **Q4** | **Q5** | **Q6** | **Q7** | **Q8** | **Q9** |
| --- | --- | --- | --- | --- | --- | --- | --- | --- | --- |
| (Mao et al., 2021) | No | No | Yes | Yes | Yes | Yes | Yes | Yes | Yes |
| (Alan et al., 2022) | No | No | Yes | Yes | Yes | Yes | Yes | Yes | Yes |
| (Hong et al., 2022) | No | No | Yes | Yes | Yes | Yes | Yes | Yes | Yes |
| (Jang et al., 2022) | Yes | No | Yes | Yes | Yes | Yes | Yes | Yes | Yes |
| (Karnjuš et al., 2021) | Unclear | No | No | Yes | Yes | Yes | Yes | Yes | Yes |
| (Li et al., 2021) | Yes | No | Yes | Yes | Yes | Yes | Yes | Yes | Yes |
| (Song et al., 2021) | No | Yes | Yes | Yes | Yes | Yes | Yes | Yes | Yes |
| (McGarity et al., 2022) | No | No | Unclear | Yes | Unclear | No | Yes | No | Yes |

**Table A 5** JBI critical appraisal checklist for quasi-experimental studies

| **Domain** | **Indicator** |
| --- | --- |
| Q1-Is it clear in the study what is the ‘cause’ and what is the ‘effect’ (i.e. there is no confusion about which variable comes first)? | Yes-No-Unclear-Not applicable |
| Q2-Were the participants included in any comparisons similar? | Yes-No-Unclear-Not applicable |
| Q3-Were the participants included in any comparisons receiving similar treatment/care, other than the exposure or intervention of interest? | Yes-No-Unclear-Not applicable |
| Q4-Was there a control group? | Yes-No-Unclear-Not applicable |
| Q5-Were there multiple measurements of the outcome both pre and post the intervention/exposure? | Yes-No-Unclear-Not applicable |
| Q6-Was follow up complete and if not, were differences between groups in terms of their follow up adequately and analyzed? | Yes-No-Unclear-Not applicable |
| Q7-Were the outcomes of participants included in any comparisons measured in the same way? | Yes-No-Unclear-Not applicable |
| Q8-Were outcomes measured in a reliable way? | Yes-No-Unclear-Not applicable |
| Q9-Was appropriate statistical analysis used? | Yes-No-Unclear-Not applicable |

**Table A 6** Quality assessment of quasi-experimental studies

|  | **Q1** | **Q2** | **Q3** | **Q4** | **Q5** | **Q6** | **Q7** | **Q8** | **Q9** |
| --- | --- | --- | --- | --- | --- | --- | --- | --- | --- |
| (Slobodin et al., 2021) | Yes | Not applicable | Not applicable | Not applicable | Yes | No | Yes | Yes | Yes |
| (Zhang et al., 2021) | Yes | Yes | Yes | Yes | Yes | Yes | Yes | Yes | Yes |
| (K. A. Qureshi et al., 2002) | Yes | Not applicable | Not applicable | Not applicable | Yes | No | Yes | Unclear | Unclear |
| (Kristine A. Qureshi et al., 2004) | Yes | Yes | Yes | Yes | Yes | No | Yes | Unclear | Yes |
| (Jen et al., 2022) | Yes | Yes | Yes | Yes | Yes | Yes | Yes | Yes | Yes |

**Table A 7** JBI critical appraisal checklist for cohort studies

| **Domain** | **Indicator** |
| --- | --- |
| Q1-Were the two groups similar and recruited from the same population? | Yes-No-Unclear-Not applicable |
| Q2-Were the exposures measured similarly to assign people to both exposed and unexposed groups? | Yes-No-Unclear-Not applicable |
| Q3-Was the exposure measured in a valid and reliable way? | Yes-No-Unclear-Not applicable |
| Q4-Were confounding factors identified? | Yes-No-Unclear-Not applicable |
| Q5-Were strategies to deal with confounding factors stated? | Yes-No-Unclear-Not applicable |
| Q6-Were the groups/participants free of the outcome at the start of the study (or at the moment of exposure)? | Yes-No-Unclear-Not applicable |
| Q7-Were the outcomes measured in a valid and reliable way? | Yes-No-Unclear-Not applicable |
| Q8-Was the follow up time reported and sufficient to be long enough for outcomes to occur? | Yes-No-Unclear-Not applicable |
| Q9-Was follow up complete, and if not, were the reason to loss to follow up described and explored? | Yes-No-Unclear-Not applicable |
| Q10-Were strategies to address incomplete follow up utilized? | Yes-No-Unclear-Not applicable |
| Q11-Was appropriate statistical analysis used? | Yes-No-Unclear-Not applicable |

**Table A 8** Quality assessment of cohort study

|  | **Q1** | **Q2** | **Q3** | **Q4** | **Q5** | **Q6** | **Q7** | **Q8** | **Q9** | **Q10** | **Q11** |
| --- | --- | --- | --- | --- | --- | --- | --- | --- | --- | --- | --- |
| (Lauck et al., 2022) | Yes | Yes | Yes | Yes | Unclear | Yes | Yes | Yes | Yes | Not applicable | Yes |

**Table A 9** JBI critical appraisal checklist for qualitative research

| **Domain** | **Indicator** |
| --- | --- |
| Q1-Is there congruity between the stated philosophical perspective and the research methodology? | Yes-No-Unclear-Not applicable |
| Q2-Is there congruity between the research methodology and the research question or objectives? | Yes-No-Unclear-Not applicable |
| Q3-Is there congruity between the research methodology and the methods used to collect data? | Yes-No-Unclear-Not applicable |
| Q4-Is there congruity between the research methodology and the representation and analysis of data? | Yes-No-Unclear-Not applicable |
| Q5-Is there congruity between the research methodology and the interpretation of results? | Yes-No-Unclear-Not applicable |
| Q6-Is there a statement locating the researcher culturally or theoretically? | Yes-No-Unclear-Not applicable |
| Q7-Is the influence of the researcher on the research, and vice-versa, addressed? | Yes-No-Unclear-Not applicable |
| Q8-Are participants, and their voices, adequately represented? | Yes-No-Unclear-Not applicable |
| Q9-Is the research ethical according to current criteria or, for recent studies, and is there evidence of ethical approval by an appropriate body? | Yes-No-Unclear-Not applicable |
| Q10-Do the conclusions drawn in the research report flow from the analysis, or interpretation, of the data? | Yes-No-Unclear-Not applicable |

**Table A 10** Quality assessment of qualitative studies

|  | **Q1** | **Q2** | **Q3** | **Q4** | **Q5** | **Q6** | **Q7** | **Q8** | **Q9** | **Q10** |
| --- | --- | --- | --- | --- | --- | --- | --- | --- | --- | --- |
| (Dhal & Mohapatra, 2022) | Unclear | Yes | Yes | Yes | Yes | Unclear | Unclear | Yes | Yes | Yes |
| (Lavin et al., 2019) | Unclear | Yes | Yes | Yes | Unclear | Unclear | Unclear | Unclear | No | Yes |
